# A randomised controlled trial of matrix-assisted laser desorption ionization-time of flight mass spectrometry (MALDITOF-MS) versus conventional microbiological methods for identifying pathogens: Impact on optimal antimicrobial therapy of invasive bacterial and fungal infections in Vietnam

**DOI:** 10.1016/j.jinf.2019.03.010

**Published:** 2019-06

**Authors:** Behzad Nadjm, Vu Quoc Dat, James I. Campbell, Vu Tien Viet Dung, Alessandro Torre, Nguyen Thi Cam Tu, Ninh Thi Thanh Van, Dao Tuyet Trinh, Nguyen Phu Huong Lan, Nguyen Vu Trung, Nguyen Thi Thuy Hang, Le Thi Hoi, Stephen Baker, Marcel Wolbers, Nguyen Van Vinh Chau, Nguyen Van Kinh, Guy E. Thwaites, H. Rogier van Doorn, Heiman F.L. Wertheim

**Affiliations:** a Oxford University Clinical Research Unit, Hanoi & Ho Chi Minh City, Viet Nam; b Centre for Tropical Medicine & Global Health, Nuffield Department of Clinical Medicine, University of Oxford, Oxford, United Kingdom; c Department of Infectious Diseases, Hanoi Medical University, Hanoi, Viet Nam; d National Hospital for Tropical Diseases, Hanoi, Viet Nam; e Hospital for Tropical Diseases, Ho Chi Minh City, Viet Nam; f Department of Medical Microbiology, RadboudUMC, Nijmegen, The Netherlands

**Keywords:** Matrix-assisted laser desorption-ionization mass spectrometry, Microbiological techniques, Bacteraemia, Antibacterial agents, Vietnam

## Abstract

**Objectives:**

We assessed the impact of MALDITOF-MS on the timeliness of optimal antimicrobial therapy through a parallel-arm randomised controlled trial in two hospitals in Vietnam.

**Methods:**

We recruited patients with a pathogen (bacterial or fungal) cultured from a normally sterile sample. Samples were randomly assigned (1:1) to identification by MALDITOF-MS or conventional diagnostics. The primary outcome was the proportion on optimal antimicrobial therapy within 24 h of positive culture, determined by a blinded independent review committee. Trial registered at ClinicalTrials.gov (NCT02306330).

**Results:**

Among 1005 randomised patients, pathogens were isolated from 628 (326 intervention, 302 control), with 377 excluded as likely contaminants or discharged/died before positive culture. Most isolates were cultured from blood (421/628, 67.0%). The proportion receiving optimal antimicrobial therapy within 24 h (the primary outcome) or 48 h of growth was not significantly different between MALDITOF-MS and control arms (135/326, 41.4% vs 120/302, 39.7%; Adjusted Odds ration (AOR) 1.17, *p* = 0.40 and 151/326, 46.3% vs 141/302, 46.7%; AOR 1.05 *p* = 0.79, respectively).

**Conclusions:**

MALDITOF-MS, in the absence of an antimicrobial stewardship programme, did not improve the proportion on optimal antimicrobial therapy at 24 or 48 h after first growth in a lower-middle income setting with high rates of antibiotic resistance.

## Highlights

• MALDITOF MS provided more rapid identification of invasive bacterial and fungal pathogens than conventional microbiology.
• MALDITOF MS did not increase the proportion of patients on optimal therapy at 24 or 48 hours after positive culture.
• MALDITOF MS did not increase the proportion of patients receiving adequate therapy at 24 hours after positive culture.
• The most common reason for therapy being sub-optimal was use of overly broad spectrum or unnecessary multiple antibiotics.

## Introduction

High quality laboratory diagnostics play an important role in the management of infectious diseases.^1^^,^^2^ Low- and middle-income countries (LMICs) often lack the resources for these. The consequence is limited knowledge of bacterial epidemiology and susceptibility, exacerbating inappropriate antibiotics use and impacting on both antimicrobial resistance (AMR) and patient outcomes.

Recognising this, investment in hospital laboratory infrastructure and capacity building in LMICs has attracted international attention.^3^, ^4^^–^^5^ Efficient use of limited available resources is needed to develop optimal laboratory capacity, avoid inappropriate use of antibiotics and improve patient outcomes.^6^ Novel technologies have been developed to improve identification and susceptibility testing results, but many are expensive^1^^,^^7^ and developed in and for high income countries^8^ but are now being introduced in LMIC laboratories. Systematic evaluation of these is important, especially in resource constrained settings, to show impact on clinical decision-making and patient care.

Matrix-assisted laser desorption ionization-time of flight mass spectrometry (MALDITOF-MS) accurately identifies cultured bacteria and fungi within minutes.^9^^,^^10^ Whilst the hardware is expensive (approximately 250,000 USD) it has very low per assay costs (1-1.5 USD/sample) and requires minimal skills.^9^^–^^11^ Reagents have long expiry dates, unlike traditional biochemical identification systems (leading to regular use of expired reagents is common in LMICs).^12^ Thus MALDITOF-MS has potential to improve microbiological diagnostics in LMICs.

Previous studies in high-income settings, where MALDITOF has been combined with an antimicrobial stewardship programme (ASP), have shown clinical impact on reduced time to optimal antibiotic therapy,^13^^,^^14^ increased proportion of appropriate antibiotic treatment after culture positivity^15^ and reduced length of hospital stay.^14^^,^^16^^–^^18^

To date no randomised controlled trials have been reported exploring the benefit of MALDITOF-MS compared with conventional microbiology in relation to clinical endpoints, nor have there been any studies of its clinical (as opposed to diagnostic) utility in LMICs. We aimed to determine whether MALDITOF-MS reduced the time to optimal antibiotic therapy compared to conventional microbiological identification in patients with confirmed infection.

## Materials and Methods

### Study design and participants

A parallel arm randomised controlled trial was conducted in 2 tertiary infectious diseases hospitals in Vietnam: the National Hospital for Tropical Diseases (NHTD) in Hanoi and the Hospital for Tropical Diseases (HTD) in Ho Chi Minh City. Both have ISO15189 accredited microbiology laboratories. Positive blood cultures or aspirates from sterile compartments (cerebrospinal fluid (CSF), deep abscesses, joint fluid, peritoneal fluid, pleural fluid or deep tissue biopsies) were randomised. Patients with at least one pathogenic bacteria or fungus cultured from such samples were recruited, patients whose cultures showed contamination were not recruited as optimal therapy for such patients would depend on the clinical picture rather than the blood culture result. Patients were not recruited if at the time of randomisation they already had an eligible sample processed during the same hospital admission.

### Microbiological methods

Clinical specimens were collected and cultured according to standard practice. Blood culture bottles (aerobic) were incubated for up to five days in an automated system (Bactec, Becton-Dickinson, USA). Other samples were incubated on media allowing growth of aerobic, anaerobic and fastidious organisms and checked daily. Positive specimens were randomly allocated to identification by either MALDITOF-MS (Microflex LT/SH, Bruker, Germany, library DB4613) or conventional diagnostics. For the MALDITOF-MS arm, positive blood culture media or colonies from plates were sub-cultured onto blood agar until growth could be observed. (Supplementary Fig. 1) Colonies were then analysed by MALDITOF-MS twice daily, a result was considered positive identification if it gave a score ≥2.00. In the control arm, methods for identification included: Gram-staining, API test strips, VITEK2 (bioMérieux, Marcy l’Étoile, France) and other tests as per standard operating procedures. All media were commercially sourced.

**Fig. 1:**
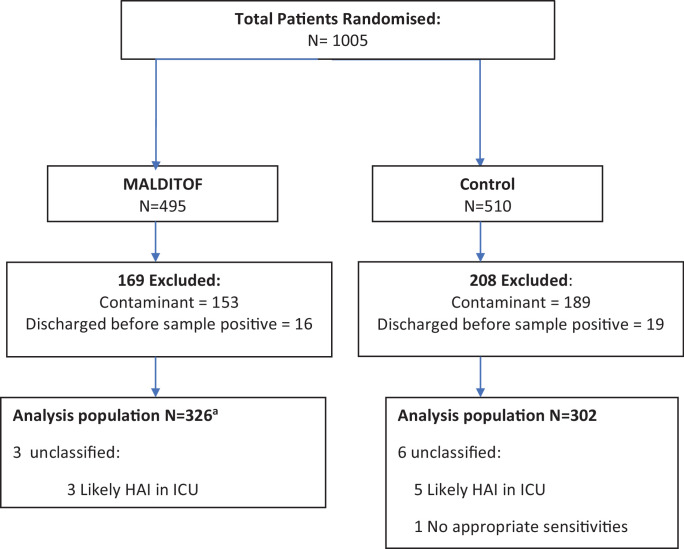
Trial flow. ^a^2 patients randomised to MALDITOF had identification by routine methods. They were analysed as per their randomisation arm (i.e. included in MALDITOF group).

### Randomization and masking

Randomisation was 1:1 by a web based randomization program using a random variable block length of 4 or 6, with stratification by hospital and specimen type (blood vs. other). When an eligible specimen showed growth, the technician entered patient and specimen code into the randomization program. The diagnostic pipeline allocation was then generated and logged. All subsequent positive eligible specimens from that patient were assigned to the same arm. Patients were recruited when the sample grew a pathogen. In HTD only this followed consent of the participant or legal representative. Samples that yielded organisms not considered pathogens (e.g. coagulase-negative staphylococci, diphtheroids, viridans group streptococci in the absence of a matching clinical syndrome) were not included. Treating physicians were not informed of the allocated arm.

### Procedures

Clinical and microbiological data were collected prospectively onto a Case Record Form (CRF) and checked for accuracy by research staff. At least 24 h following delivery of the written report to the ward, the research team asked the clinical team if this had changed management and if not, why not.

No other changes were made to routine hospital procedures for communication of culture results to clinical teams. This involved direct reporting of Gram-stains and positive culture results by phone followed by issue of a written report through the internal postal system. No antimicrobial stewardship intervention was involved in the study. Hospital staff had access to a variety of international and national guidelines.

### Outcomes

The primary outcome was the proportion of patients on optimal antimicrobial treatment within 24 h of positive culture (first observed growth of an eligible specimen). Optimal antimicrobial treatment was defined as treatment using any drug to which the isolate showed *in vitro* susceptibility (using AST results from the same agent or by proxy according to CLSI guidelines) and had known clinical susceptibility, but not including unnecessarily broad spectrum regimens. The decision on optimal therapy was made by an independent review committee, blinded to the diagnostic arm. The committee reviewed the admission and discharge diagnosis, the antimicrobials used during the admission and the full microbiology results. The committee were asked to determine the following: presence of optimal antimicrobial therapy at 24 h and at 48 h (secondary endpoint), or at any point during hospital stay. If therapy was not optimal at 24 h the reasons were grouped into: too broad, organism not covered, and ‘other’. Examples of ‘too broad therapy’ would include: use of a carbapenem for treatment of confirmed meningitis or sepsis due to *Streptococcus suis* or *Streptococcus pneumoniae* or non-ESBL producing, cephalosporin susceptible *Enterobacteriaceae*; use of a combination of ß-lactam and another agent for treatment of *Enterobacteriaceae* susceptible to the ß-lactam *etc*. Inter-reviewer discrepancies were resolved by discussion of the case and review of international guidelines. If the committee considered the organism was not the only reason for antimicrobial therapy a decision of ‘unclassifiable’ was recorded.

### Statistical analysis

We expected the proportion of patients on optimal therapy within 24 h to increase from 40% (conventional diagnostics) to 60% (MALDITOF-MS).^15^ To detect this with 90% power at the 2-sided significance level of 5%, requires a total sample size of 260. To allow sufficient power for a subgroup analysis in blood cultures for each hospital separately, the target enrolment was 280 patients recruited at the slower recruiting hospital.

The statistical analyses were predefined in an analysis plan. For the primary outcome we used a logistic regression model of the primary outcome depending on arm, with additional adjustment for the first specimen type (blood vs. other) and study site. As a conservative measure, subjects with an ‘unclassifiable’ primary outcome were labelled as ‘non-optimal’, as were subjects that were discharged or died within 24 h unless optimal therapy had been started before death/discharge. Subgroup analyses and secondary outcome of optimal therapy within 48 h of positive culture were performed in the same way as for the whole population. Analyses were performed using R (Version 3.4.0). P values below 0.05 were considered significant (two-sided). Further details available in supplementary material (statistics analysis plan).

### Ethics

Eligible patients received written information about the study, informing them of its purpose, procedures, their right to refuse participation and how to get more information or withdraw. Any patient who requested not to be enrolled had their specimens labelled accordingly and diagnostics as per routine practice.

The institutional review board (IRB) in the National Hospital for Tropical Diseases (77/HDDD-NDTU) approved the study without the need for individual patient consent. The IRB in the Hospital for Tropical Diseases (16-HDDD-QD) required all patients (or legal representatives) to be seen by study staff, informed of the study, and give written consent before participation. The study was also approved by the Oxford Tropical Research Ethics Committee (55-14) and registered at ClinicalTrials.gov (NCT02306330).

## Results

### Study Population

The trial recruited between 1st December 2014 and 15th January 2016. The study was stopped when the sample size for the primary outcome was exceeded. 1005 patients with a positive sterile site culture were randomized. In accordance with the protocol, the 342 cultured bacteria considered contaminants and the 35 drawn from patients who had either died or been discharged by the time the culture became positive were excluded post randomisation, leaving 628 patients for the analysis (Fig. 1). Among the 628 samples, 421 (67.0%) were blood, 154 (24.5%) CSF, 46 (7.3%) peritoneal fluid, 6 (1.0%) deep abscess samples, and 1 (0.2%) pleural fluid. 635 bacterial or fungal isolates were obtained (1 patient had 4 isolates in a single culture and 4 patients had 2 isolates). There were 105 fungi, including *Cryptococcus neoformans* (63/635, 9.9%) and *Talaromyces marneffei* (36/635, 5.7%). There were no significant differences in baseline variables between the two arms (Tables 1 and 2).

**Table 1:** Baseline characteristics of groups by patients.

	*N*	MALDITOF-MS n (% or IQR)	*N*	Control n (% or IQR)	P*
Sex	326		302		0.45
- Female		121 (37)		103 (34)	
Age (median years)		47 (32–59)		47 (35–58)	0.91
Site	326		302		0.57
- Ho Chi Minh city (HCMC)		187 (57)		180 (60)	
- Hanoi		139 (43)		122 (40)	
Source	325		302		0.58
- Direct Admission		163 (50)		144 (48)	
- Hospital transfer		162 (50)		158 (52)	
Ward	326		302		0.41
- Critical Care		79 (24)		82 (27)	
- Other		247 (76)		220 (73)	
Site of infection	326		302		0.33
- Central nervous system (CNS)		81 (25)		89 (29)	
- Abdominal		83 (25)		81 (27)	
- Respiratory		31 (10)		23 (8)	
- Other		40 (12)		44 (15)	
- Unknown		91 (28)		65 (22)	
ICD-10 Code	325		302		0.12
- Sepsis		87 (27)		81 (27)	
- HIV related		69 (21)		59 (20)	
- CNS Infection		46 (14)		49 (16)	
- Cirrhosis		28 (9)		29 (10)	
- Tetanus		6 (2)		4 (1)	
- Other		89 (27)		80 (27)	
Length of illness (median days)	321	6 (3–14)	300	6 (3–14)	0.62
Time from sample collection to first growth (median hours)		34 (22–45)		36 (22–46)	0.41
Time from sample collection to Gram stain (median hours)	287	31 (21–43)	267	33 (20–44)	0.63
Specimen type	326		302		0.61
- Blood Culture		222(68)		199 (66)	
- Other		104 (32)		103 (34)	
Pathogen type	326		302		0.40
- Gram-positive		103 (32)		111 (37)	
*Streptococcus suis*		38 (12)		46 (15)	
-Gram-negative		167 (51)		137 (46)	
*Escherichia coli*		83 (25)		64 (21)	
- Fungi		52 (16)		52 (17)	
- Mixed		4 (1)		2 (1)	

⁎ Fisher's exact test for proportions and Kruskal–Wallis test for non-parametric data.

**Table 2:** Baseline characteristics by organisms isolated.

	*N*	MALDITOF-MS n (%)	*N*	Control n (%)	P*
Identification	329		306		
Gram-negative					
Total *Enterobacteriaceae*		131 (40)		104 (34)	0.15
- *Escherichia Coli*		83 (25)		64 (21)	
- *Klebsiella pneumoniae*		29 (9)		21 (7)	
- Other *Enterobacteriaceae*		19 (6)		19 (6)	
Total Non-*Enterobacteriaceae*		40 (12)		36 (12)	0.98
- *Acinetobacter* & *Pseudomonas* spp.		14 (4)		12 (4)	
- Other Gram-negatives		26 (8)		24 (8)	
Total Gram-positive		105 (32)		114 (37)	0.18
- Streptococci		70 (21)		85 (28)	
- *Staphylococcus aureus*		32 (10)		26 (8)	
- Other Gram-positives		3 (1)		3 (1)	
Total Fungi		53 (16)		52 (17)	0.85
- *Cryptococcus neoformans*		29 (9)		34 (11)	
- *Talaromyces marneffei*		20 (7)		16 (5)	
- Other fungi		4 (1)		2 (1)	
Bacteria resistance profiles (where tested)					
- *S. aureus* with methicillin resistance	32	16 (50)	26	15 (58)	0.99
- *Enterobacteriaceae* with 3G-C resistance	131	63 (48)	103^a^	50 (49)	1
- *Enterobacteriaceae* with carbapenem resistance	123^b^	9 (7)	97^c^	1^1^	0.06
- *Acinetobacter* or *Pseudomonas* species with carbapenem resistance	13^d^	5 (38)	11^e^	5 (45)	1
- *Enterococci* with vancomycin resistance	5	1 (20)	6	2 (33)	1

⁎ Fisher's exact test. 3G-*C* = 3rd generation cephalosporin.

a 1 isolate not tested (*K. pneumoniae*).

b 8 isolates not tested (*1 Klebsiella pneumoniae*, 7 *Salmonella* spp.).

c 7 isolates not tested (2 *E. coli*, 2 *K. pneumoniae*, 3 *Salmonella* spp.).

d 1 isolate not tested (*Acinetobacter sp.*).

e 1 isolate not tested (*Acinetobater baumannii*).

### Primary outcome

The proportion of patients who received optimal therapy within 24 h, was not different between MALDITOF-MS (135/326, 41.4%) and control arms (120/302, 39.7%) (Adjusted Odds Ratio (AOR) 1.17; 95% confidence interval (CI) 0.82–1.67, *p* = 0.40). In 9 cases (3 MALDITOF-MS, 6 control arm) the review committee recorded therapy as ‘unclassifiable’ and these were included in the ‘not optimal’ outcome as per the analysis plan. The predominant reason for the committee to consider a treatment non-optimal was because therapy was too broad (254/373, 68.1%) (Table 3).

**Table 3:** Reasons for non-optimal therapy at 24 h after culture growth according to the independent review committee.

	MALDITOF-MS	Control
	*N* = 191	*N* = 182
	*n* (%)	*n* (%)
Pathogen not covered	54 (28.3)	46 (25.3)
Therapy too broad	130 (68.1)	122 (67)
Therapy potentially effective but not ideal	3 (1.6)	6 (3.3)
Growth of second pathogen within 48 h that was not covered	1 (0.5)	2 (1.1)
No data	3 (1.6)	6 (3.3)

### Secondary outcomes

**Fig. 2:**
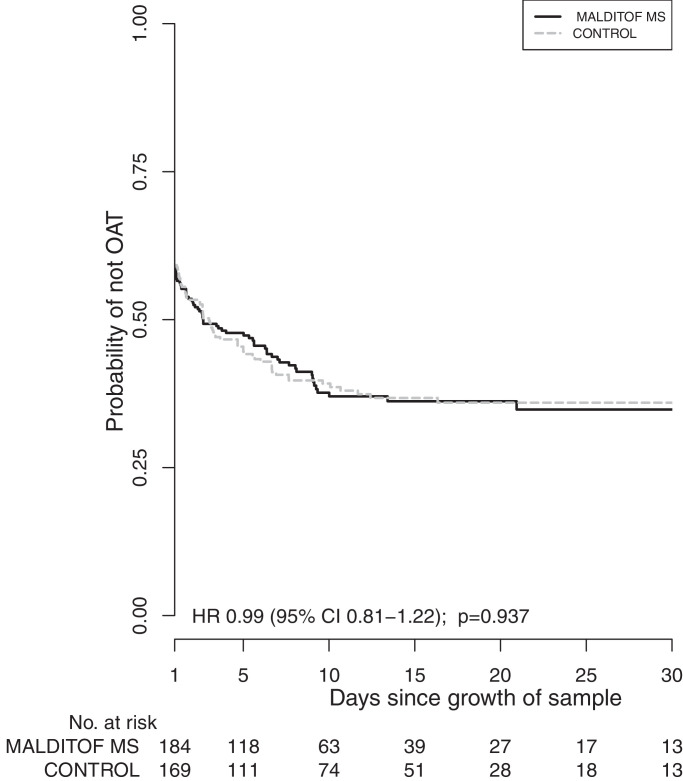
Time from growth to optimal antimicrobial therapy (OAT).

There was no difference in the proportion of patients on optimal therapy within 48 h of growth between MALDITOF-MS (151/326, 46.3%) and control arms (141/302, 46.7%, AOR 1.05 *p* = 0.79) (Table 4) or in the time from growth to optimal antimicrobial therapy (HR 0.99 (95%CI 0.81– 1.22) *p* = 0.937) (Fig. 2).

**Table 4:** Proportions of patients on optimal antibiotic therapy within 24 and 48 h of growth.

	*N*	MALDITOF-MS *n* (%)	*N*	Control *n* (%)	AOR (95% CI)^a^	*p*
Within 24 h	326	135 (41.4)	302	120 (39.7)	1.17 (0.82–1.67)	0.40
Within 48 h	326	151 (46.3)	302	141 (46.7)	1.05 (0.74–1.50)	0.79

a Adjusted odds ratio adjusted for specimen type (blood/other) and site.

There was no difference in the ordinal outcome (hospital outcome grouped into 5 categories - death, palliative discharge, survived with sequelae, transferred to another hospital and recovered) adjusted for site and sample type, between the MALDITOF-MS and control arms (AOR 0.869 (95%CI 0.65 – 1.16) *p* = 0.34). Although median hospital stay was the same for both arms, Cox proportionate hazards adjusted for site and specimen type demonstrated an increased hazard ratio for hospital discharge in the MALDITOF-MS arm (Table 5 & Supplementary Fig. 2). Analysis in survivors only showed similar median length of stay in the MALDITOF-MS (15 days, IQR 11-21) and control arms (16 days, IQR 11-23). There was no significant difference in other pre-specified secondary outcomes (Table 5 and Supplementary Fig. 3).

**Table 5:** Pre-specified secondary outcomes.

	*N*	MALDITOF-MS	*N*	Control	*P*
DDD of antimicrobial consumption from enrolment to discharge (median, IQR)	304	16.0 (8.4–33)	283	18.0 (9.0–39.3)	0.35^a^
Days of antimicrobial therapy from enrolment to discharge (median, IQR)	304	10 (6–15)	282	11 (7–17.8)	0.287^b^
Days in hospital (Median, IQR)	322	15 (10–21)	301	16 (10–23)	0.039^c^
Days spent in Critical Care (Median, IQR)	115	5 (2–11)	100	4 (3–10.3)	0.38^d^
Hours from first growth to pathogen identification (Median, IQR)	324	2.2 (1.7–28.1)	298	26.6 (24.7–48)	ND
Hours from sample collection to pathogen identification (Median, IQR)	324	43.2 (28.1–69.1)	298	66 (48–88.6)	ND
Days from sample collection to hospital discharge (Median, IQR)	326	11 (6.1–16.3)	302	12.3 (7.2–19)	ND

a Linear regression coefficient 0.92 (95% CI 0.78–1.09) after adjustment for site and specimen type.

b Hazard ratio for stopping antibiotics 1.09 (95% CI 0.92–1.29) after adjustment for site and specimen type.

c Hazard ratio for hospital discharge 1.18 (95% CI 1.01–1.38) after adjustment for site and specimen type.

d Hazard ratio for ICU discharge 1.13 (95% CI 0.86–1.49) after adjustment for site and specimen type in those that had an ICU stay ND statistical comparison not performed (as stipulated in the analysis plan).

### Subgroup analyses

Limiting the analysis to the subgroup of patients with Gram-positive organisms cultured showed a trend towards an increased proportion on optimal therapy at 24 h in the MALDITOF-MS arm (45/103, 43.7%) compared to the control arm (41/111, 36.9%; *p* = 0.1). However, there was no significant effect observed in any pre-specified subgroup analysis (Table 6).

**Table 6:** Pre-specified subgroup analyses of proportion of patients on optimal therapy within 24 and 48 h of culture growth in predefined subgroups.

	Proportion optimal within 24 h of culture growth						Proportion optimal within 48 h of culture growth					
	*N*	MALDITOF *n* (%)	*N*	Control n (%)	AOR (95% CI)^a^	*P*	*N*	MALDITOF *n* (%)	*N*	Control *n* (%)	AOR (95% CI)^a^	*P*
Sample Type						0.43^b^						0.58^b^
- Blood	222	75 (33.8)	199	59 (29.6)	1.31 (0.84–2.07)	0.23	222	88 (39.6)	199	76 (38.2)	1.14 (0.73–1.78)	0.56
- Other	104	60 (57.7)	103	61 (59.2)	0.96 (0.53–1.72)	0.88	104	63 (60.6)	103	65 (63.1)	0.91 (0.50–1.65)	0.75
Site						0.60^b^						0.98^b^
- HCMC	187	108 (57.8)	180	101 (56.1)	1.09 (0.72–1.67)	0.68	187	122 (65.2)	180	116 (64.4)	1.05 (0.68–1.62)	0.83
- Hanoi	139	27 (19.4)	122	19 (15.6)	1.38 (0.71–2.74)	0.34	139	29 (20.9)	122	25 (20.5)	1.06 (0.57–1.99)	0.86
Pathogen type						0.61^b^						0.51^b^
- Gram-positive	103	45 (43.7)	111	41 (36.9)	1.74 (0.90–3.42)	0.10	103	47 (45.6)	111	47 (42.3)	1.42 (0.74–2.74)	0.30
- Gram-negative	167	58 (34.7)	137	47 (34.3)	1.09 (0.65–1.82)	0.75	167	70 (41.9)	137	62 (45.3)	0.91 (0.55–1.52)	0.72
- Fungi	52	31 (59.6)	52	32 (61.5)	1.09 (0.41–3.01)	0.86	52	33 (63.5)	52	32 (61.5)	1.38 (0.52–3.70)	0.53
Admitted from						0.90^b^						0.72^b^
- Home	163	71 (43.6)	144	61 (42.4)	1.17 (0.72–1.90)	0.53	163	80 (49.1)	144	71 (49.3)	1.10 (0.68–1.79)	0.70
- Hospital	162	64 (39.5)	159	59 (37.3)	1.16 (0.68–1.99)	0.59	162	71 (43.8)	158	70 (44.3)	0.99 (0.58–1.69)	0.97
Final diagnosis						0.73^b^						0.76^b^
- Meningitis	76	57 (75.0)	65	48 (73.8)	1.28 (0.56–3.00)	0.56	76	59 (77.6)	65	51 (78.5)	1.11 (0.46–2.66)	0.81
- Other	250	78 (31.2)	237	72 (30.4)	1.07 (0.71–1.63)	0.74	250	92 (36.8)	237	90 (38.0)	0.96 (0.64–1.45)	0.86

a Adjusted for site and specimen type except where these are part of the subgroup.

b Test for heterogeneity.

### Exploratory analyses

An analysis of mortality as a binary outcome (death or palliative discharge compared with all other outcomes) adjusted for site and sample type revealed no difference (MALDITOF-MS arm 52/326 (16.0%), control arm 43/302 (14.2%), AOR 1.13 (95%CI 0.73–1.76, *p* = 0.59)).

Excluding outliers (the longest staying 1% of patients) in an analysis of hospital stay to explain the increased hazards ratio for hospital discharge in the MALDITOF-MS arm, the hazard ratio for discharge in the MALDITOF-MS arm dropped to 1.16 (*p* = 0.067).

An analysis to determine whether results were reaching the wards more quickly in the MALDITOF-MS arm demonstrated that the median time from growth to the pathogen identification report being received on the ward was 10.1 h (IQR 1.9 – 32.9 h) in the MALDITOF-MS arm and 31.0 h (IQR 27.4–54 h) in the control arm.

A subgroup analysis of the 146 patients not on optimal therapy at the time of culture growth, that subsequently did receive optimal therapy, showed that the median time to optimal therapy was 2.0 (IQR 0.7–5.6) days in the MALDITOF-MS arm and 2.6 (IQR 1.2 – 4.8) days in the control arm. A further subgroup analysis of those patients according to whether they were in critical care or not when cultures were drawn showed no significant difference in both those in critical care (OR 0.90 (95% CI 0.5 – 1.72, *p* = 0.81)) or other wards (OR 1.16 (95%CI 0.79–1.69, *p* = 0.45)).

An analysis looking at the proportion receiving antibiotic therapy at 24 h after culture growth that lacked *in vitro* activity against the isolated pathogen (inadequate therapy) showed little difference between the two arms (54/326, 16.6% and 46/302, 15.2% in MALDITOF-MS and control arms respectively).

## Discussion

Our study demonstrates that early identification of pathogens from cultures of blood and other sterile sites using MALDITOF-MS did not result in a difference in the proportion of patients on optimal therapy within 24 h of first growth. Neither did MALDITOF-MS alter the proportion on optimal therapy by 48 h, the time taken to provide optimal therapy, the duration or total antibiotic therapy, patient outcomes or time in intensive care. We found an association between MALDITOF-MS and earlier hospital discharge, but the significance was removed when outliers were excluded (very long stay patients) and we consider it unlikely to be clinically significant. Ours is not the first study to demonstrate that technological advances in rapid diagnostics, though compelling, do not always lead to improvements in clinically relevant outcomes in the absence of an antibiotic stewardship programme. A similar result was found in a randomized study of the impact of peptide nucleic acid fluorescence in situ hybridisaton (PNA-FISH) on a variety of clinical outcomes in a tertiary care hospital in the USA.^19^

In common with previous studies, we found quicker pathogen identification and reporting. Our study gives some indication as to why MALDITOF-MS results did not result in improved outcomes. In both arms Gram stain results for positive blood cultures were available rapidly and possibly already provided sufficient information. The most common cause of suboptimal therapy was use of excessively broad therapies, suggesting that there were delays or reluctance in de-escalation of therapy. There was some evidence that the intervention was more successful in patients with Gram-positive infections. This may relate to identifying *Streptococcus suis*, a common cause of both meningitis and severe sepsis which has as yet not evolved reduced susceptibility to penicillin^20^ and exclusion of alternative pathogens.

One other trial of MALDITOF-MS compared with conventional microbiology with 28 day mortality as the primary endpoint has completed recruitment in the UK but has yet to be reported (the RAPIDO trial, https://doi.org/10.1186/ISRCTN97107018). While other studies have established that MALDITOF-MS can identify pathogens in the tropics,^21^ all eight publications that explored the clinical impact of MALDITOF-MS^13^^–^^18^^,^^22^^,^^23^ were conducted in high income countries (HICs). Three explored the impact of MALDITOF-MS compared with conventional diagnostics^15^^,^^16^^,^^22^ without an ASP component. One was restricted to peritoneal dialysis fluid,^16^ the others recruited patients with bloodstream infections.^15^^,^^22^ One showed a significant improvement in the proportion with appropriate therapy within 24 h of growth (from 64% to 75.3%, *p* = 0.01),^15^ while the other found a non-significant improvement in the proportion receiving active treatments within 48 h of blood cultures being (from 89.8% to 95.6%, *p* = 0.09).^22^ Five studies examined the impact of MALDITOF-MS plus ASP with conventional diagnostics without ASP.^13^^,^^14^^,^^17^^,^^18^^,^^23^ These studies all showed improvements in time to active or appropriate therapy and two showed lower mortality.^13^^,^^14^

Our study has the advantage of addressing the single intervention of MALDITOF-MS, highlighting the need to investigate additional supports, such as ASP, to achieve clinical impact. It cannot be generalized to settings where ASP are already in place. The individually randomised nature of the study is robust but this design may not account for changes in prescribing that could arise from a ‘cultural shift’ resultant from a wholesale change in diagnostic practice. Although the use of two sites and the large sample size is a strength, the use of specialist infectious diseases hospitals could cause bias and poor generalisability. However, it seems unlikely that MALDITOF-MS alone would be more effective at changing prescribing practice in a setting where staff are less experienced in managing infection and the uniformity of the results across different pathogen groups makes it unlikely that the case mix seen is responsible for the negative results. There may be criticism over the subjectivity of the primary endpoint (optimal therapy as determined by a panel of experts) and others have utilized spectrum-of-activity scores to demonstrate improvements in de-escalation.^24^^,^^25^ However, the blinded nature of the committee, and the use of a single committee for all evaluations, should have minimized bias. Additionally, the absence of benefit in the comparison of the proportion receiving inadequate therapy in the two arms at 24 h suggests that the findings were real. We did not collect data on patient severity (SOFA/APACHE II scores), making it difficult to assess whether a subgroup of either more or less severe patients may have seen benefit from the intervention. However an exploratory analysis showed no effect of the intervention in patients that were in critical care at the time cultures were drawn. Our study did not achieve the prespecified sample size. However, this large sample size was determined to accurately assess if the intervention was effective for blood cultures in each hospital, we surpassed the sample size necessary for the primary outcome and it is thus unlikely we missed a relevant positive result. Our setting has particularly high proportions of antibiotic resistant organisms, and results may not be generalisable to settings where these are lower. Although the contamination rate was noted to be high during the study, it is not outside that reported in the literature.^26^ Attempts were made to reduce the contamination rate through additional education for those responsible for venepuncture, and replacement of liquid disinfection fluids with disposable sterile alcohol wipes. There was a small number of cases where the endpoint review committee was unable to reach a decision (6 in the control arm and 3 in the MALDITOF-MS arm), these results could not have changed the result of the primary outcome (data not shown).

Despite these negative findings there are several positive aspects to MALDITOF-MS that should not be overlooked. Firstly, even speedier identification can be achieved by both processing samples direct from blood culture^27^ (without the subculture onto blood agar) and by running the machine more frequently than twice daily. However, this requires changes to work flow that were not possible within the trial and, based on the results we obtained, would be unlikely to have had an impact on the results. Rapid AST, either through short incubation with antibiotics or through analysis of the spectra obtained, has also now been described using MALDITOF-MS.^28^ Though more technically difficult, such results may have been more compelling in this setting and warrant further evaluation.

In conclusion, our study showed no improvement in antimicrobial prescribing or other patient or provider centred outcomes through MALDITOF-MS, though MALDITOF-MS did produce results rapidly in our setting. While MALDITOF-MS has many other compelling advantages, our findings suggest that it is unlikely to lead to improvements in prescribing on its own. Further studies in this setting exploring the addition of ASPs, and education of the diagnostic and prescribing workforce would be useful.

## Appendix Supplementary materials

Image, application 1

Image, application 2
