# Supplementary material for: A randomised controlled trial of matrix-assisted laser desorption ionization-time of flight mass spectrometry (MALDITOF-MS) versus conventional microbiological methods for identifying pathogens: Impact on optimal antimicrobial therapy of invasive bacterial and fungal infections in Vietnam
Source: J Infect. 2019 Jun;78(6):454–60. doi: 10.1016/j.jinf.2019.03.010 (PMC6529875; doi:10.1016/j.jinf.2019.03.010)
Supplement: Supplementary file 1 [file mmc1.docx]

**Supplementary Figure 1.** Flow chart of microbiological methods

**
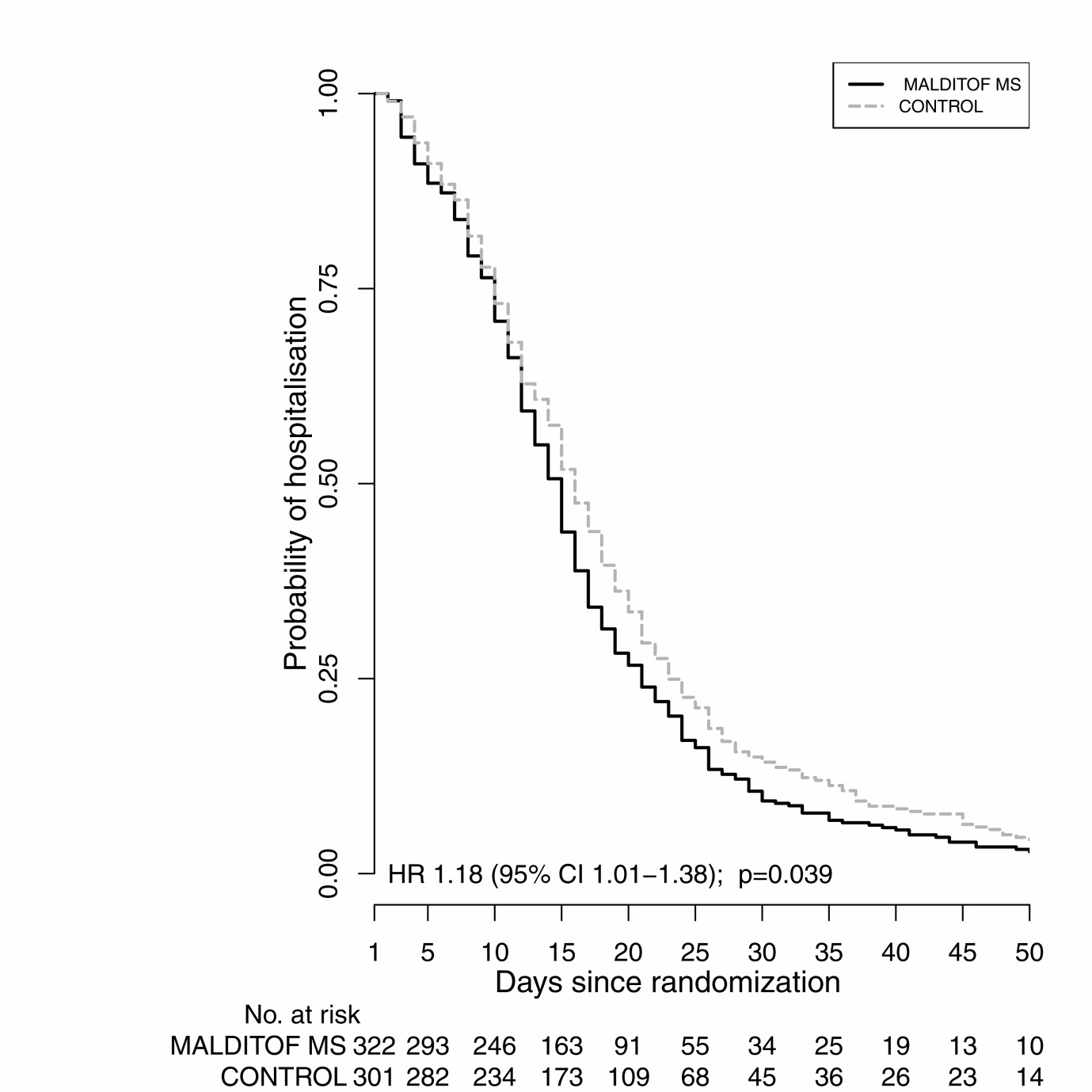
**

**Supplementary Figure 2.** Time from patient sampling to optimal antimicrobial therapy (OAT)


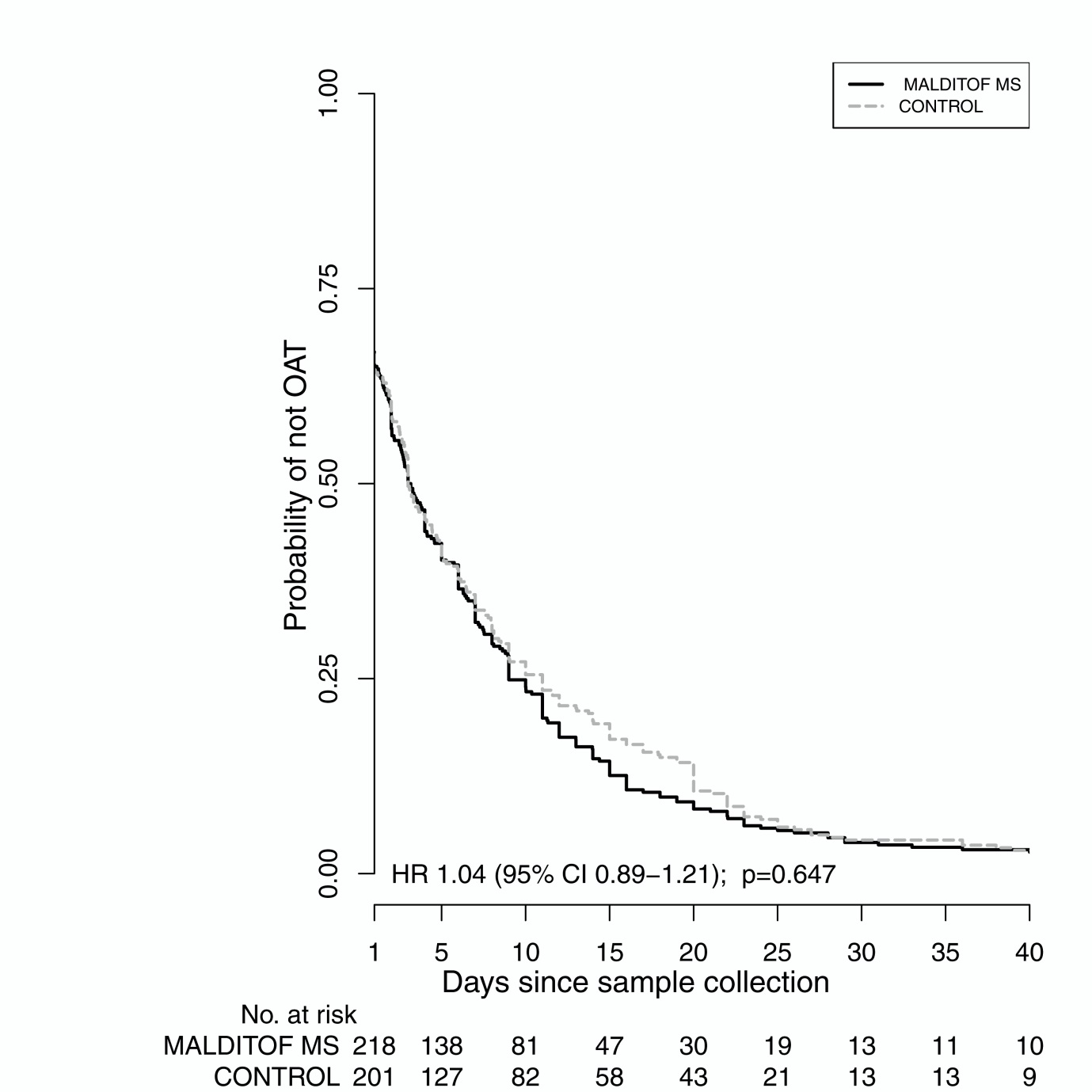


**Supplementary Figure 3.** Time to hospital discharge
