# Supplementary material for: A randomised controlled trial of matrix-assisted laser desorption ionization-time of flight mass spectrometry (MALDITOF-MS) versus conventional microbiological methods for identifying pathogens: Impact on optimal antimicrobial therapy of invasive bacterial and fungal infections in Vietnam
Source: J Infect. 2019 Jun;78(6):454–60. doi: 10.1016/j.jinf.2019.03.010 (PMC6529875; doi:10.1016/j.jinf.2019.03.010)
Supplement: Supplementary file 2 [file mmc2.docx]

# Statistical analysis plan for A randomised controlled trial of matrix-assisted laser desorption ionization–time of flight mass spectrometry (MALDITOF-MS) versus conventional microbiological methods for identifying pathogens: impact on optimal antimicrobial therapy of invasive bacterial and fungal infections in Vietnam (NCT02306330)

Authors: Dung Vu Tien Viet, Heiman Wertheim, Behzad Nadjm

# **Purpose**

This document details the planned analyses and endpoint derivations for the 09HN study. It focuses on the analyses for the main clinical paper.

# **Statistical software**

The data derivations and statistical analyses will be performed with the statistical software R using the current R version at the time of the final analysis.

# **Analysis population and definition of baseline**

The main population for all analyses is the population of all subjects for which the primary endpoint could be defined by the endpoint review committee (modified intention-to-treat). All analyses will be according to the randomized diagnostic arm.

The following participant will be excluded from the modified intention-to-treat population:

- Patients who discharged or died before the culture flagged positive (DISCHARGE.DischargeDate + DISCHARGE.DischargeTime before SAMPLE.GDetectedDate + SAMPLE.GDetectedTime).
- Patients where the endpoint review committee felt the culture was not in itself enough to guide therapy (e.g. potential contaminant, viridans strep in a patient without signs of endocarditis),.

Baseline is defined as the time point when the participant’s first culture flagged positive.

All patients excluded from the modified intention to treat population and reasons for exclusion will be summarized by diagnostic arm (“MALDITOF MS” or “ROUTINE DIAGNOSTICS”).

# **Baseline characteristics**

We use Chi-square for interaction test for heterogeneity.

Baseline characteristics will be summarized by the randomized diagnostic arm as median (lower and upper quartile) for continuous data and percentage for categorical data.

The following baseline characteristics will be summarized:

- Gender
- Age
- Enrolment hospital
- Enrolment ward will be classified as “Critical care” vs. “other”
- Was the patient coming from home or transferred from another hospital?
- Duration of this illness at hospital admission
- Major site of the infection leading to randomization
- Final patient diagnosis (ICD10 code) [note: this is not strictly a baseline variable]
- First sample type: detailed enrolment specimen and specimen classified as blood vs. other
- Time to growth enrolment specimen
- Detected organism in enrolment specimen: Gram (+), Gram (-), mixed, fungi – this will be manually coded from the organism arm

During the drafting of the first draft of the manuscript it was decided that it was necessary to add the following:

- Time to Gram stain
- Bacteriology and resistance profiles (to include MRSA, 3^rd^ generation cephalosporin resistance in enterobateriaceae, carbapenem resistance, vancomycin resistant enterococci)

# **Primary endpoint: proportion of patients on optimal antibiotic treatment within 24 hours of positive culture (first growth of an eligible specimen)**

## **Derivation**

A patients is defined to be on optimal antibiotic treatment within 24h of positive culture (first growth of an eligible specimen) according to the endpoint committee.

As a conservative measure, patients in the modified intention-to-treat population who withdrew or died within 24 hours of positive culture will be classified as not being on optimal antibiotic treatment by 24 hours unless the optimal antibiotic treatment was already started while the subject was still in the study and alive, similarly those where the endpoint committee judged that they could not classify the outcome as the organism was not the only reason for antimicrobial therapy will be classified as not being on optimal therapy.

## **Planned analysis**

The number (%) of patients receiving the optimal antibiotic treatment within 24 hours of positive culture will be described by diagnostic arm overall and in pre-defined subgroups (see below).

For the formal comparison between the two diagnostic groups, we will use a logistic regression model of the primary endpoint depending on the treatment groups, with additional adjustment for the first specimen type (blood vs. other) and the hospital. The main effect measure is an odds ratio of optimal antibiotic treatment within 24 hours between treatment groups and a corresponding two-sided 95% confidence interval (CI) and p-value. P-values ≤0.05 are considered significant.

The analysis will be implemented using the R function glm.

The reasons why subjects were not on optimal antibiotics therapy within 24 hours will be summarized descriptively as frequency of reasons (%) amongst patients without optimal treatment.

## **Subgroup analyses**

The analysis of the primary outcome will also be performed in the following pre-defined subgroups:

- First specimen type: blood / other
- Hospital
- Type of pathogen in first culture: Fungal, bacterial, or other (mixed)
- Type of bacteria in first culture (amongst those with bacteria detected in the culture): Gram positive / Gram negative
- Admitted from home/transferred from another hospital
- Final diagnosis: Meningitis /other final diagnosis

Treatment effects in subgroups will be estimated in the same way as for the whole population, i.e. subgroup analyses will also be adjusted by specimen group and hospital unless the adjustment variable is part of the subgroup definition. Heterogeneity of the treatment effect across subgroups will be tested using likelihood ratio tests for an interaction between the treatment assignment and the respective sub-grouping variable.

# **Secondary endpoint**

## **Optimal therapy within 48hrs of positive culture**

This endpoint will be analysed in the same way as the primary endpoint.

Where the endpoint is Unknown and the patient was discharged <24 hours after admission, the endpoint should be changed considered not optimal unless recorded as optimal.

## **The total amount of prescribed antibiotics to the patients from enrolment until hospital discharge in defined daily doses (DDD)**

**Derivation**

The defined daily dose (DDD) is a statistical measure of drug consumption, defined by the World Health Organization (WHO), which can be obtained from the WHO Antimicrobial DDD Quick Reference List.

The total amount of prescribed antibiotics (in DDD) is defined as the sum of the DDD’s assigned to each administered drug. The number of DDD’s for each administered drug can be calculated based on the drug name, dose, route, frequency, and the number of days this drug was administered. This information is available in the dataset.

As drugs consumption after hospital discharge was not formally collected, the drug end date will be imputed with the discharge date in case the end date is missing.

**Planned analysis**

The total amount of prescribed antibiotics per patients will be summarized as median (lower and upper quartile). Comparisons between the groups will be based on a linear regression model with treatment as the main covariate and adjustment by the first specimen type and hospital. In case the outcome has a pronounced right skewed distribution, the endpoint will be log-transformed prior to the analysis leading to a comparison of geometric rather than arithmetic means. (the outcome was skewed and therefore log transformed)

As for patients who died early, the amount of prescribed antibiotics is low despite their poor outcome, the endpoint will also be summarized (and informally compared) in survivors only.

## **The total duration of antibiotics and antifungals treatment from enrolment until hospital discharge**

**Derivation**

The total duration of antibiotics (including antifungals) will be derived based on the DRUGS log. As administration of different antibiotics might overlap in time or there may be gaps between different administration episodes, the derivation will first derive start and stop dates of each “antibiotics episode”, i.e. non-overlapping time period where at least one antibiotic was administered. The total duration is then the sum of the duration of all episodes of the subject. As drugs consumption after hospital discharge was not formally collected, the drug end date will be imputed with the discharge date in case the end date is missing.

**Planned analysis**

The duration of antibiotic treatment will be visualized using Kaplan-Meier curves and estimate of median (IQR) of durations in both groups.

Formal comparisons between the two diagnostic arms will be based on the Cox proportional hazards model with the diagnostic arm as the only covariate and stratification by hospital and first specimen type. As for patients who died early, the duration of antibiotic treatment is low despite their poor outcome, the endpoint will also be summarized (and informally compared) in survivors only.

## **Hospital and ICU length stay**

**Derivation**

The duration of hospital stay in days is defined as

Discharge Date – Admission Date +1

The duration of ICU stay is the sum of all stays in ICU wards (including potential ICU re-admissions). The start data of an ICU episode is either the hospital admission day (if the patient was directly admitted to ICU) or the transfer date to an ICU ward. The day of ICU discharge is either the transfer date to the next non-ICU ward or (if no further transfers occur) the hospital discharge date.

**Planned analysis**

The duration of hospital and ICU length stay will be visualized using Kaplan-Meier curves and estimate of median (IQR) of durations in both groups.

Formal comparisons between the two treatment arms will be based on the Cox proportional hazards model with the diagnostic arm as the only covariate and stratification by hospital and first specimen type. As for patients who died early, the duration of hospitalisation or ICU stay is low despite their poor outcome, the endpoint will also be summarized (and informally compared) in survivors only.

## **Outcome: death or palliative discharge, survived with sequelae, recovered**

**Derivation**

As for patients who were transferred to other hospital, the discharge status will be grouped with sequelae at discharge.

**Planned analysis**

The frequency of the discharge outcome will be summarized by diagnostic arm. The ordinal outcome will be compared between the two diagnostic arms based on a proportional odds cumulative logit model with the diagnostic arm as the main covariate and adjustment by hospital and first specimen type.

## **The time from first growth of an eligible specimen to optimal antibiotic treatment**

**Derivation**

Duration between the two events = Date-time of optimal treatment – Date-time of first growth of an eligible specimen.

Patients who received optimal antibiotics treatment will have the event type “received optimal antibiotics treatment”. Patients who did not receive documented optimal antibiotics treatment will be considered as having a competing event “death without prior optimal treatment” at their death date (if they died prior to receiving optimal treatment) or a competing event “discharge/other without optimal treatment” at their hospital discharge date.

**Planned analysis**

The cumulative incidence of subjects starting “optimal antibiotics treatment”, or experiencing “death without prior optimal treatment” or “discharge/other without optimal treatment”, respectively will be visualized by diagnostic group using cumulative incidence functions.

The cumulative incidence of being on optimal antibiotics treatment will be compared between the two arms based on a Fine and Gray competing risks model with treatment as the main covariate and stratification by first specimen group and hospital. As there is no censoring in the dataset, i.e. all subjects experience an event type, this can be implemented with a standard Cox regression model where patients with competing events are treated as right-censored at time “infinity” to indicate that they never received optimal antibiotics treatment. Of note, this analysis is different from the analysis outlined in the protocol which specified a cause-specific model but seems more appropriate and consistent with the analysis of the primary endpoint.

In addition, the median (IQR) time to optimal antibiotics treatment will be summarized in the subset of subjects who eventually receive optimal antibiotics treatment only.

## **The time from collection of first positive eligible specimen to optimal antibiotic treatment**

This endpoint will be derived and analyzed in the same way as the time from first growth of an eligible specimen to optimal antibiotic treatment except that time “0” is now the time of the first specimen collection.

## **Time from first growth of an eligible specimen to issue of pathogen identification report, time from first specimen collection to issue of pathogen identification report, and time from first specimen collection to discharge**

These three time-to-event endpoints will be descriptively summarised by diagnostic arm as median (lower and upper quartiles) in patients for whom the outcome is evaluable only and no formal comparison between the groups will be performed.

The time to the issue of a pathogen identification report is only evaluable in subjects for whom the identified pathogen reported to the ward or doctor is non-missing.

## **Other pre-planned analyses**

- The number of participants in which the identification report did not lead to a change in antibiotic/antifungal treatment on the day of receiving the report will be summarized by diagnostic arm.
- The reasons given by the ward for not streamlining treatment at the time provided on the day of receiving the identification report is also collected. Nine different reasons (including “other”) are specified but doctors are allowed to tick multiple reasons. As an initial analysis, the frequency of a ‘yes’ answer for each of the nine reasons (yes/no for each reason) will be summarized separately by treatment arm. In a second step, the frequency of all possible combinations of reasons that were ticked by doctors will be summarized.

## The following extra analyses were added on 13/9/17

Mortality as a binary outcome – inpatient death + patients given palliative discharge vs other

Time from culture positive to time report received on ward.

Analyse those not given appropriate therapy (ie not optimal and reason is not covered) at 24 hours and compare between arms.

Summarise reasons for non-optimal therapy in each arm
